# Effects of nitrogen nutrition on the synthesis and deposition of the ω-gliadins of wheat

**DOI:** 10.1093/aob/mct291

**Published:** 2013-12-15

**Authors:** Yongfang Wan, Cristina Sanchis Gritsch, Malcolm J. Hawkesford, Peter R. Shewry

**Affiliations:** Department of Plant Biology and Crop Science, Rothamsted Research, Harpenden, Hertfordshire AL5 2JQ, UK

**Keywords:** Wheat, *Triticum aestivum*, storage protein, nitrogen, ω-gliadin, RNA *in situ* hybridization, immunolocalization, protein bodies, wheat allergy

## Abstract

**Background and Aims:**

The ω-gliadin storage proteins of wheat are of interest in relation to their impact on grain processing properties and their role in food allergy, particularly the ω-5 sub-group and wheat-dependent exercise-induced anaphylaxis. The ω-gliadins are also known to be responsive to nitrogen application. This study therefore compares the effects of cultivar and nitrogen availability on the synthesis and deposition of ω-gliadins in wheat grown under field conditions in the UK, including temporal and spatial analyses at the protein and transcript levels.

**Methods:**

SDS–PAGE, western blotting and N-terminal amino acid sequencing were used to compare the patterns of ω-gliadin components in mature grain of six British wheat (*Triticum aestivum*) cultivars and their accumulation during the development of grain grown in field plots with varying nitrogen supply. Changes in gene expression during development were determined using real-time reverse transcription–PCR (RT–PCR). Spatial patterns of gene expression and protein accumulation were determined by *in situ* hybridization and immunofluorescence microscopy, respectively.

**Key Results:**

Two patterns of ω-gliadins were identified in the six cultivars, including both monomeric ‘gliadin’ proteins and subunits present in polymeric ‘glutenin’ fractions. Increasing the level of nitrogen fertilizer in field plots resulted in increased expression of ω-gliadin transcripts and increased proportions of ω-5 gliadins. Nitrogen supply also affected the spatial patterns of ω-gliadin synthesis and deposition, which were differentially increased in the outer layers of the starchy endosperm with high levels of nitrogen.

**Conclusions:**

Wheat ω-gliadins vary in amount and composition between cultivars, and in their response to nitrogen supply. Their spatial distribution is also affected by nitrogen supply, being most highly concentrated in the sub-aleurone cells of the starchy endosperm under higher nitrogen availability.

## INTRODUCTION

Wheat is the most important food crop in the temperate world, being used to produce bread, pasta, noodles and a range of other baked goods and foods. The ability to produce this wide range of products is largely determined by the grain storage proteins (prolamins), which form a viscoelastic network, called gluten, in dough formed from wheat flour.

In common with other groups of seed storage proteins, the wheat prolamins are highly polymorphic, being encoded by multigene families with homoeoallelic genes present on the three genomes (A, B and D) of bread wheat. There is also extensive allelic variation between the gluten proteins present in different genotypes. The wheat prolamins are classically divided into two groups: the gliadins which are monomeric proteins and contribute to dough viscosity and extensibility, and the polymeric glutenins which contribute to dough elasticity (strength). Within these groups, the individual proteins are further classified by their electrophoretic mobility, the gliadins into α-type, γ-type and ω-gliadins on the basis of their mobility on electrophoresis at low pH, and the glutenin sub-units into high molecular weight (HMW) and low molecular weight (LMW) groups based on their separation by SDS–PAGE (Shewry *et al.*, 2009*a*).

The classification into gliadins and glutenins has proved to be remarkably durable, but does not reflect the true molecular and evolutionary relationships of the proteins. Based on these, only three groups can be recognized (Shewry *et al.*, 1986): the HMW prolamins (comprising the HMW subunits of glutenin), the sulfur-rich (S-rich) prolamins (comprising the α-, β- and γ-gliadins and the LMW subunits of glutenin) and the S-poor prolamins which comprise the ω-gliadins and related proteins present in the glutenin fraction (called the D group of LMW subunits) (Masci *et al.*, 1993, 1999).

Most of the ω-gliadins and related proteins (from hereon all referred to as ω-gliadins) are encoded by genes at the *Gli-1* loci on the short arms of chromosomes A, B and D (called *Gli-A1*, *Gli-B1* and *Gli-D1*), although minor additional loci on the same chromosome arms have been reported (reviewed by Tatham and Shewry, 2012). There is also a clear distinction between the structures and properties of the proteins encoded by *Gli-A1* and *Gli-D1* and those encoded by *Gli-B1.* Although both groups of proteins consist mainly of sequence repeats based on short peptide motifs, these motifs differ, being based on PQQPFPQQ in the proteins encoded by *Gli-A1* and *Gli-D1* and PFQ_2–4_ in the proteins encoded by *Gli-B1* (where P is proline, Q is glutamine and F is phenylalanine). These differences in sequence are reflected in the amino acid compositions of the whole proteins, with the ω-gliadins encoded by *Gli-A1* and *Gli-D1* comprising about 40 mol% glutamine and 30 mol% proline and those encoded by *Gli-B1* comprising about 50 mol% glutamine and 20 mol% proline. Furthermore, these two types of ω-gliadin are readily separated by electrophoresis at low pH, with the *Gli-A1* and *Gli-D1* proteins which migrate more slowly being termed ω-1/2 gliadins and the *Gli-B1* proteins which migrate faster being termed ω-5 gliadins (reviewed by Shewry *et al.*, 2009*a*). The ω-gliadins can be distinguished by their N-terminal amino acid sequences, which are SRLLSPQ in ω-5 gliadins, ARQLNPSNKELQ or KELQSPQQS in ω-1 gliadins and ARELNPSNK in ω-2 gliadins (Shewry *et al.*, 2009*a*).

The ω-5 gliadins are of particular interest because they have been identified as the major components responsible for triggering wheat-dependent exercise-induced anaphylaxis (WDEIA) in susceptible individuals. This allergenic response occurs when wheat is ingested before physical exercise and the symptoms are unusually acute for wheat allergy and can lead to death. Several studies have shown that ω-5 gliadins are the major allergens in WDEIA (Palosuo *et al.*, 2001; Morita *et al.*, 2003) and identified immunodominant epitopes in the repeated sequence domain (Battais *et al.*, 2005; Matsuo *et al.*, 2005).

The ω-gliadins have been the least well studied of all groups of wheat prolamins, due to their relatively low abundances, lack of well established relationships with technological properties, and technical difficulties in characterizing both the proteins and their genes (resulting from their highly repetitive structures). Nevertheless, it is clear that they show levels of genetic polymorphism that are similar to those found in other groups of gluten proteins (Metakovsky, 1991; Denery-Papini *et al.*, 2007). The ω-gliadins generally account for about 10–20 % of the total gliadins (Wieser *et al.*, 1994), but the proportion is increased by either increased nitrogen availability or sulfur deficiency (Moss *et al.*, 1981, 1983; Hurkman *et al.*, 2013), with the former resulting in increased transcript levels as well as greater protein accumulation (Altenbach and Kothari, 2007).

We have therefore carried out a detailed analysis of the ω-gliadin fractions in six cultivars of wheat grown in the UK, in order to determine their properties and expression patterns both in the developing grain and in response to applied nitrogen fertilizer. This not only increases our knowledge of this fascinating group of proteins but also provides a basis for future attempts to manipulate their amount and composition by breeding, agronomy or processing.

## MATERIALS AND METHODS

### Plant materials and harvest

Six bread wheat (*Triticum aestivum*) cultivars, ‘Cordiale’, ‘Hereward’, ‘Istabraq’, ‘Marksman’, ‘Malacca’ and ‘Xi19’, were grown in field trials with three replicate blocks at Rothamsted Research (Harpenden, UK) in 2009 and 2010 (for agronomic details, see Barraclough *et al.*, 2010). Nitrogen was applied at three levels (100, 200 and 350 kg N ha^−1^, referred to as N100, N200 and N350) before anthesis. Main stem ears were tagged at anthesis and whole caryopses were harvested from the middle part of the ear at 14, 21, 28 and 35 days post-anthesis (DPA) and frozen immediately in liquid nitrogen. The four time points correspond to the start (14 DPA), middle (21 DPA) and end (28 DPA) of the main grain-filling period and the start of grain maturation and desiccation (35 DPA) (Shewry *et al.*, 2009*b*).

### RNA extraction and RT–PCR

RNA was extracted from frozen caryopses using a method based on Chang *et al.* (1993). About 1·5 g of whole caryopses were ground in a cooled mill and extracted with CTAB buffer [2 % (w/v) cetyltrimethyl ammonium bromide (CTAB), 2 % (w/v) polyvinyl pyrrolidine (PVP) K30, 100 mm Tris–HCl, pH 8·0, 25 mm EDTA, 2·0 m NaCI, 0·5 g L^−1^ spermidine, 2 % (w/v) 2-mercaptoethanol] with chloroform:isoamyl alcohol (IAA) (24:1) to remove proteins. RNA was precipitated by 10 m LiCl and incubation overnight on ice, dissolved in SSTE buffer [1·0 m NaCl, 0·5 % (w/v) SDS, 10 mm Tris–HCl, pH 8·0, 1 mm EDTA] to remove polysaccharides and extracted once with chloroform:IAA. After ethanol precipitation, total RNA was dissolved in diethylpyrocarbonate (DEPC)-treated water and stored at –80 °C.

For reverse transcription–PCR (RT–PCR), total RNA was cleaned with a mini RNeasy RNA isolation kit (Qiagen) and treated with RNase-free TURBO DNase (Ambion). A 5 µg aliquot of total RNA was used for reverse transcription with SuperScript^™^III reverse transcriptase (Invitrogen) using anchored oligo(dT)_23_ primers (Sigma-Aldrich). cDNA diluted 1:10 was used for RT–qPCR in a 25 µL reaction with 1× SYBR Green PCR master mix (Invitrogen). Three technical and three biological replicates were analysed for each time point. RT–qPCR was performed on an ABI 7500 Real Time PCR system (Applied Biosystems).

The transcript Ta.2526.1.S1_at was used as an internal control, as it showed the most stable expression in developing caryopses of ‘Hereward’ between 6 and 42 DPA (Wan *et al.*, 2008, 2013). Primers for RT–qPCR and the synthesis of *in situ* probes were designed using Primer-3 software, based on the C-terminal and 3′-untranslated region sequences of accession numbers AB181300 and GH727235 for ω-5 gliadin and of AF280605 and BQ838934 for ω-2 gliadin. They are shown in Supple-mentary Data Table S1. The specificity of the primers was verified by the amplification of single bands resolved on 3 % (w/v) agarose gels and by dissociation melting curves. The efficiencies of PCR of ω-2 and ω-5 primers were estimated as 101·8 and 100·7 %, respectively, using the LinRegPCR software (Ramakers *et al.*, 2003). The relative expression was calculated by using 7500 sequence detection software version 1.4 (Applied Biosystems) in the formula ratio = 2^−ΔΔCt^.

### Protein extraction

Wheat flour was prepared by milling in a ball mill and sieving to remove bran particles. Total protein was extracted from 20 mg of flour in 400 µL of gel loading buffer [50 mm Tris–HCI, pH 6·8, 2 % (w/v) SDS, 10 % (v/v) glycerol, 2 % (w/v) dithiothreitol (DTT) and 0·1 % (w/v) bromophenol blue]. Total gluten proteins were extracted with 50 % (v/v) aqueous propan-1-ol containing 2·5 % DTT at 50 °C, the extraction being repeated and the supernatants combined, freeze-dried, and dissolved in gel loading buffer.

For western blotting, monomeric gliadin proteins were extracted in 50 % (v/v) aqueous propan-1-ol twice at 50 °C with shaking for 30 min. The supernatant after centrifugation was dried in an Eppendorf Concentrator 5301 and resuspended in loading buffer without DTT. The pellet was resuspended in loading buffer to extract total residual proteins including polymeric glutenin proteins as reduced subunits.

The samples were heated at 90 °C for 3 min and centrifuged for 15 min at 13 000 rpm. Aliquots of the supernatants (10 µL) were separated on pre-cast 4–12 % Bis-Tris Nu-PAGE gels (Invitrogen) for western blotting. The gels were stained in Coomassie BBR250 in 10 % (w/v) trichloroacetic acid (TCA), 40 % (v/v) methanol, and destained in 10 % (w/v) TCA.

### Western blotting

For western blotting, the proteins were transferred onto mini-nitrocellulose membrane using iBlot Gel Transfer Stacks (Invitrogen) following the manufacturer's instructions. The blot was rinsed briefly in TBS (Tris-buffered saline) and then blocked in TBST [TBS with 0·05 % (v/v) Tween-20] containing 1 % (w/v) BSA (bovine serum albumin) for 1 h. The membrane was incubated with the primary antibody (diluted 1:5000) for 2 h followed by the secondary antibody (either anti-mouse or anti-rabbit alkaline phosphatase conjugated at 1:6250 dilution) for 1 h after two washes in TBS. The blot was washed in developing buffer (0·1 m Tris, 0·1 m NaCl, 50 mm MgCI_2_) and developed with NBT (nitro blue tetrazolium chloride)/BCIP (5-bromo-4-chloro-3-indolyl phosphate, toluidine salt) developing solution (Sigma-Aldrich) for a few minutes. Images were obtained using a HPG4010 scanner.

The primary antibodies used for identification of ω-gliadins were: two rabbit polyclonal antibodies and one mouse monoclonal antibody, raised against the N-terminal sequence (SRLLSPRGKELGC) of ω-5, and one rabbit polyclonal antibody raised against the N-terminal sequence (ARELNPSNKELGC) of ω-2 gliadins (Denery-Papini *et al.*, 1994, 2000).

### Gel scanning and analysis

Gels were scanned using an HPG4010 scanner and the images from grey tiff files were processed using Phoretix 1D advanced software (Nonlinear Dynamics, Durham, NC, USA). Dilution series of total gluten protein preparations were initially analysed, allowing a concentration to be selected which was within the linear response region for all ω-gliadin bands. The volume of each protein band was extracted after background subtraction using the rolling ball method. The proportions of individual ω-gliadins were calculated as the percentage of the total ω-gliadin group.

### Sample fixation and embedding

Endosperm parts of fresh grains of ‘Hereward’ at 17 and 27 DPA were cut into 2 mm transverse sections and immediately fixed in 4 % (w/v) paraformaldehyde in 0·1 m Sorenson's phosphate buffer (NaH_2_PO_4_·2H_2_O and Na_2_HPO_4_·12H_2_O, pH 7·0) for *in situ* hybridization and with 2·5 % (w/v) glutaraldehyde for immunofluorescence labelling. Tissues incubated on ice were infiltrated under vacuum three times for 10 min each and then stored at 4 °C overnight. After dehydration in increasing concentrations of ethanol and HistoClear, the sections were embedded in paraffin (Paraplast Plus, Sigma-Aldrich) for *in situ* hybridization or infiltrated with LR White Resin for several days at room temperature and polymerized at 55 °C for immunofluorescence.

Wax-embedded grains were sectioned at 12 µm thickness using a Leica JUNG Biocut 2035 rotary microtome, and resin-embedded grains were sectioned at 1 µm thickness using a Reichert-Jung Ultracut ultramicrotome. Sections were floated in water on a hot plate at 40 °C, collected on slides coated with poly-l-lysine hydrobromide, and dried at 37 °C overnight. Protein bodies were stained with 1 % (w/v) Naphthol Blue Black in 7 % (w/v) acetic acid.

Three wax- or resin-embedded grains from each treatment were sectioned, and 8–16 sections from each grain were tested for immunofluorescence or *in situ* hybridization.

### Preparation of RNA probes for *in situ* hybridization

A T7 polymerase site sequence (underlined in GAATTGTAATACGACTCACTATAGGG) was added to the 5' ends of the primers to make sense or antisense probes (Supplementary Data Table S1). PCR products of 180–217 bp were first amplified, and then *in vitro* transcribed using a Dig RNA labelling Kit (Digoxigenin-UTP, Roche). The transcribed RNA was immediately hydrolysed in 100 mm carbonated buffer at 60 °C for 20 min, and precipitated in sodium acetate and ethanol overnight at –25 °C. The RNA pellet was dissolved in DEPC-treated water, and a 1:100 dilution was used for hybridization.

### Hybridization and washing

The wax sections were deparaffinized in HistoClear, rehydrated in a decreasing ethanol series, digested with proteinase K for 30 min at 37 °C and post-fixed in 4 % paraformaldehyde in PBS (phosphate-buffered saline, pH 7·4) for 10 min. The sections were acetylated for 10 min in 0·1 m triethanolamine buffer with 0·5 % acetic anhydride, dehydrated in an ethanol series, and hybridized with the digoxigenin (DIG)-labelled RNA probes at 50 °C overnight. After post-hybridization washes in 1× SSC (saline sodium citrate buffer) and NTE buffer (0·5 m NaCl, 1 mm Tris–HCl, 2 mm EDTA) at 50 °C, the tissues were treated with RNase A at 37 °C for 30 min and, after further stringent washes, they were incubated in 1 % blocking reagent (Roche) for 2 h. The sections were incubated with anti-DIG alkaline phosphatase antibody conjugate (Roche) diluted at 1:1600 at 4 °C overnight. After washing in TBS and colour developing buffer (0·1 m Tris, 0·1 m NaCl, 50 mm MgCl_2_), the colour was developed in developing buffer with 10 % (w/v) PVA (polyvinyl alcohol, Sigma-Aldrich) and NBT/BCIP (Roche) for a few hours. The slides were washed in water to stop the colour development, followed by increasing sequential ethanol washes, and air dried. The slides were mounted in DPX mountant and observed with a Zeiss Axiophot microscope. Images were acquired with a RetigaExi CCD digital camera (Qimaging, Surrey, BC, Canada) under bright-field optics and MetaMorph software version 7.5.5 9 (Molecular Devices, Sunnyvale, CA, USA).

### Immunofluorescence labelling

Resin sections were rinsed briefly in PBST [PBS containing 0·1 % (v/v) Tween-20] and blocked in PBST containing 3 % (w/v) BSA for 1 h. The sections were incubated in the first (ω-5 monoclonal) antibody (1:100) for 2 h at room temperature. Alexafluor 568 (red) goat anti-mouse IgG was used as the secondary antibody. After incubation in the dark for 1 h in the secondary antibody (1:200 diluted in PBST containing 1 % BSA), the sections were rinsed several times in PBST, PBS and H_2_O. The sections were examined with a Zeiss 780LSM confocal microscope. The images of whole grains were captured in Z-stack series and are displayed as maximum intensity projections. An orange/yellow colour was chosen for the whole grains to help visualization. Higher magnification images of the lobe were acquired in a single optical plane and the red fluorescence and bright-field channels were merged.

## RESULTS

### Analysis of ω-gliadins by SDS–PAGE and western blotting

**Fig. 1.**
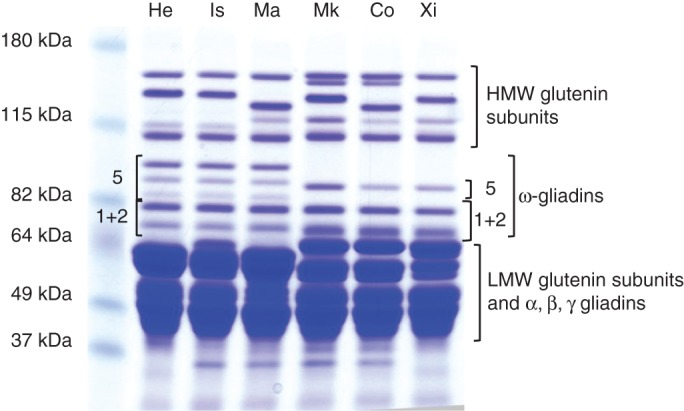
SDS–PAGE analysis of reduced total gluten protein fractions from the six cultivars: He, ‘Hereward’; Is, ‘Istabraq’; Ma, ‘Malacca’; Mk, ‘Marksman’; Co, ‘Cordiale’; Xi, ‘Xi 19’. The ω-5 and ω-1 + 2 gliadins are indicated for ‘Hereward’ and ‘Xi19’.

**Fig. 2.**
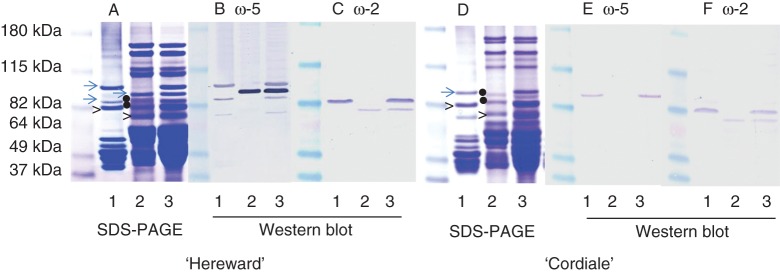
Identification of ω-gliadins in ‘Hereward’ (A–C) and ‘Cordiale’ (D–F) by SDS–PAGE (A, D) and western blot analysis with antibodies to ω-5 (B, E) and ω-2 (C, F) gliadins. The monomeric gluten proteins extracted by 50 % (v/v) propan-1-ol, reduced residual proteins (including glutenin subunits) and total proteins are shown in lanes 1, 2 and 3, respectively. The dots indicate proteins in the ω-gliadin region which did not react with either antibody and were not present in the total gluten protein fractions (Fig. 1).

SDS–PAGE separates gluten protein fractions from wheat flour into three groups based on their mobility: HMW subunits of glutenin; ω-gliadins; and LMW subunits of glutenin and other gliadins. Two patterns of ω-gliadin bands were observed in the six cultivars, comprising five bands in ‘Istabraq’, ‘Hereward’ and ‘Malacca’ but only three bands in ‘Xi19’, ‘Cordiale’ and ‘Marksman’ (Fig. 1). The ω-gliadins were initially classified as S-poor prolamins as they mostly lacked cysteine residues and were present only in monomeric form (Shewry *et al.*, 1986). However, some ω-gliadins have single cysteine residues and are present in the polymeric glutenin fraction (where they have also been called D-type LMW subunits of glutenin) (Masci *et al.*, 1993, 1999). We therefore compared the SDS–PAGE patterns of monomeric gliadin (Fig. 2A, D, lane 1) and reduced residual proteins (including glutenin subunits) (Fig. 2A, D, lane 2) by western blotting. Three antibodies specific for ω-5 gliadins (all raised against the N-terminal sequence SRLLSPRGKELGC) gave essentially similar binding patterns, and the results obtained with a single polyclonal antibody and fractions from ‘Hereward’ are shown in Fig. 2B. Four bands are labelled in the total protein extract (Fig. 2B, lane 3), three of which are present in the monomeric gliadin fraction (Fig. 2B, lane 1) and one in the reduced residual protein fraction (Fig. 2B, lane 2). The identities of the three slowest proteins as ω-5 gliadins have previously been confirmed by N-terminal amino acid sequencing of bands excised from gels (Shewry *et al.*, 2009*b*) (Supplementary Data Fig. S1A). These three proteins were therefore designated as ω-5a, ω-5b and ω-5c, respectively. The identity of the fourth band could not be confirmed by N-terminal sequencing due to the small amount of expressed protein.

Similar analyses of the fractions from ‘Hereward’ with an antibody specific for ω-2 gliadin (raised against the N-terminal sequence ARELNPSNKELGC) identified two bands in the total protein extract (Fig. 2C, lane 3), strong binding to a band which was also present in the monomeric gliadin fraction (Fig. 2C, lane 1) and weak binding to a band also present in the reduced residual protein fraction (Fig. 2C, lane 2). N-terminal amino acid sequencing of these bands has shown that both comprised a mixture of sequences of ω-1 and ω-2 gliadins (Shewry *et al.*, 2009*b*; Supplementary Data Fig. S1A). They are therefore referred to as ω-(1 + 2)a and ω-(1 + 2)b gliadins, respectively. However, the two bands differed in the proportions of ω-1 and ω-2 gliadin sequences, with ω-(1 + 2)a having a higher proportion of ω-1 sequence and ω-(1 + 2)b having equal proportions of ω-1 and ω-2 sequences. Other bands in the reduced residual protein fraction (indicated by dots in Fig. 2A, lane 2) did not react with the antibodies and did not give any sequences on Edman degradation (carried out as described by Shewry *et al.*, 2009*b*). These proteins were not present in total reduced gluten protein fractions (Fig. 1) and therefore may not be gluten proteins. The cultivars ‘Malacca’ and ‘Istabraq’ showed identical patterns of ω-gliadins on SDS–PAGE and western blotting patterns to ‘Hereward’ and are not shown.

Analysis of ‘Cordiale’ showed only single bands binding to the ω-5 antibody in the total protein (Fig. 2E, lane 3) and monomeric gliadin fractions (Fig. 2E, lane 1), with no binding to the reduced residual protein fraction (Fig. 2E, lane 2). ‘Cordiale’ had the same two ω-2 gliadin bands as ‘Hereward’, with one being monomeric [ω-(1 + 2)a] and the other polymeric [ω-(1 + 2)b] (Fig. 2F; Supplementary Data Fig. S1B). Two bands present in the ω-gliadin region of the reduced residual protein fraction (indicated by dots in Fig. 2D, lane 2) did not bind either antibody. These proteins were not present in the total gluten protein fraction (see Fig. 1) and hence were not gluten proteins. The cultivars ‘Xi19’ and ‘Marksman’ showed identical patterns of ω-gliadins on SDS–PAGE and western blotting to ‘Cordiale’ and are not shown.

### Response of ω-gliadin proteins to nitrogen

Comparison of SDS–PAGE separations of total gluten protein fractions indicated that nitrogen fertilization resulted in increases in the proportion of ω-gliadins, as reported by many other workers (Moss *et al.*, 1981; Dupont *et al.*, 2006*a*, *b*; Altenbach and Kothari, 2007; Godfrey *et al.*, 2010).

**Fig. 3.**
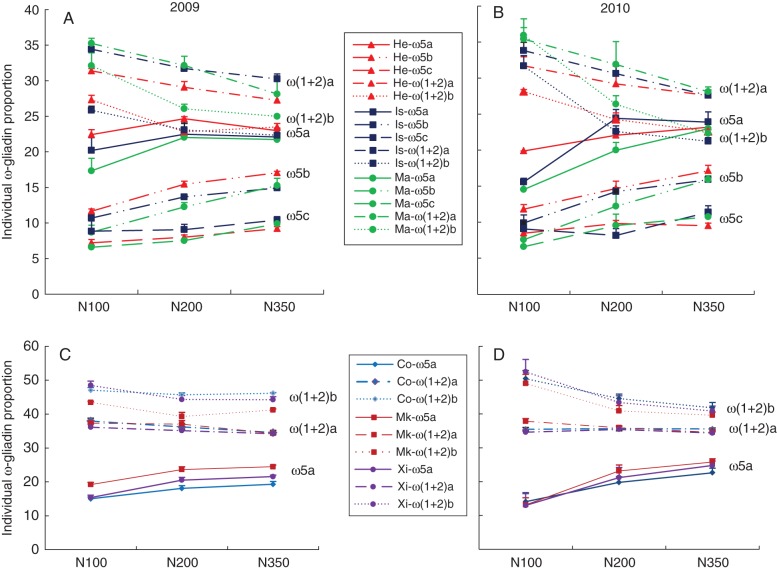
The effects of nitrogen fertilization (at 100, 200 and 350 kgN ha^−1^) on the proportions of individual ω-gliadins in grain grown in 2009 (A, C) and 2010 (B, D).

We therefore determined the effects on the proportions of individual ω-gliadin bands, by quantitative scanning of SDS–PAGE separations of total gluten protein fractions extracted using 50 % (v/v) propan-1-ol and 2·5 % (v/v) DTT. This showed clear effects of nitrogen on the relative proportions, with increases in the proportion of ω-5 gliadins and decreases in the proportions of ω-(1 + 2) gliadins (Fig. 3). This effect was consistent between the six genotypes and in the two years. Furthermore, in ‘Hereward’, ‘Istabraq’ and ‘Malacca’, the proportion of the polymeric ω-5b protein increased more than the proportions of the two monomeric ω-5 gliadins (ω-5a and ω-5c). Similarly, the proportion of the polymeric ω-(1 + 2)b band was reduced more than that of the monomeric ω-(1 + 2)a band in all six cultivars.

### Regulation of ω-gliadin gene expression by nitrogen supply

**Fig. 4.**
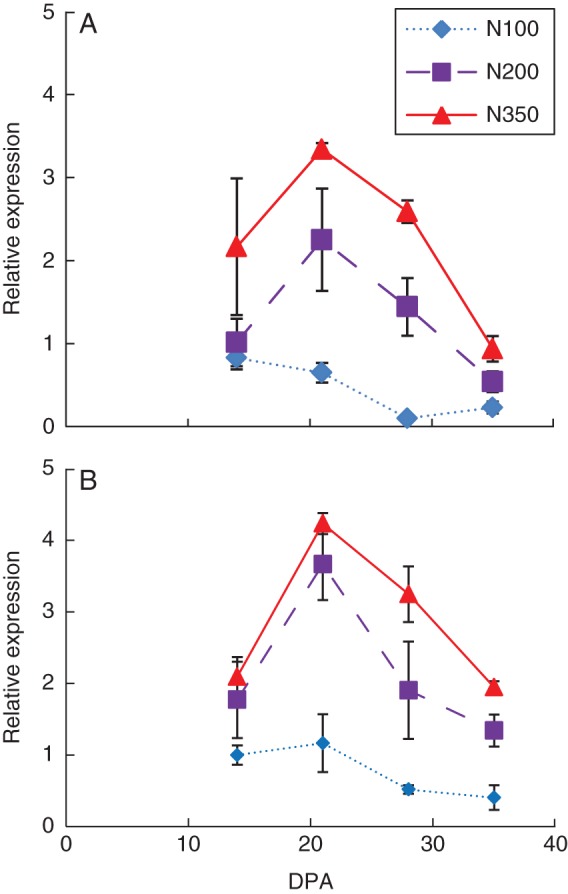
The effects of nitrogen fertilization (100, 200 and 350 kgN ha^−1^) on the expression profiles of transcripts related to (A) ω-5 and (B) ω-2 gliadins during grain filling of ‘Hereward’ grown in 2010, determined by real-time RT–PCR.

Real-time RT–PCR was used to determine ω-2 and ω-5 gliadin gene expression in ‘Hereward’ at 14, 21, 28 and 35 DPA (Fig. 4). The effects of nitrogen application on the abundances of transcripts for ω-2 and ω-5 were already apparent at 14 DPA. Transcript abundance reached a maximum at 21 DPA, being 3- to 4-fold greater at N200 and N350 compared with N100. However, the effect of nitrogen treatment was less at 35 DPA (after the main grain-filling period).

### Localization of ω-gliadin gene expression by *in situ* hybridization

**Fig. 5.**
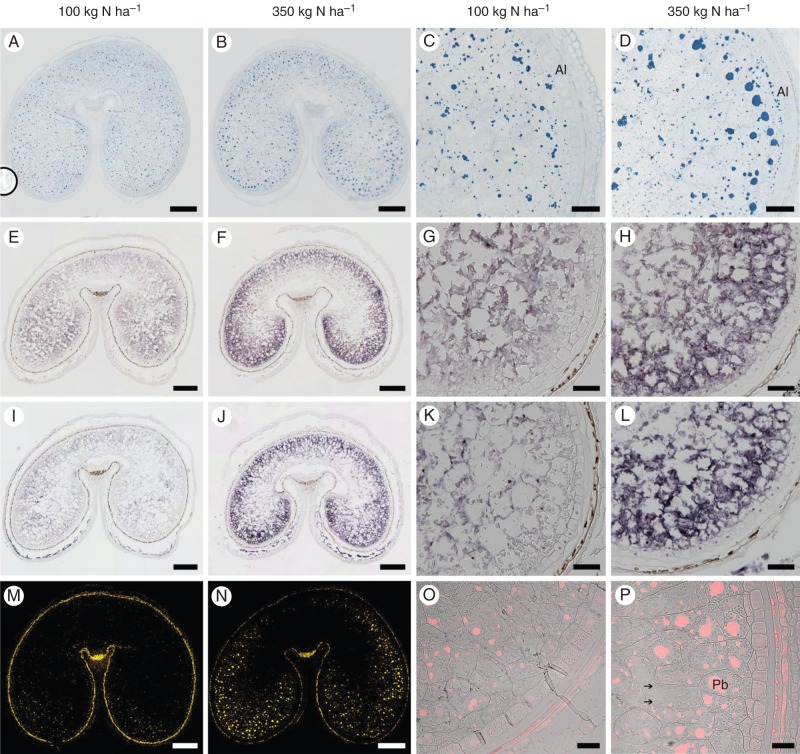
Spatial patterns of deposition of ω-gliadins in the starchy endosperm of wheat ‘Hereward’ grown at 100 or 350 kg N ha^−1^, as indicated above the columns. Whole-grain sections are shown in the first two columns, and higher magnifications of lobes are shown in the second two columns. (A–D) Sections at 27 DPA stained for protein bodies with Naphthol Blue Black, (E–H) *in situ* hybridization of transcripts related to ω-5 gliadins at 17 DPA and (I–L) *in situ* hybridization of transcripts related to ω-2 gliadins at 17 DPA. (M–P) Immunolocalization of ω-5 gliadin at 27 DPA. The immunofluorescence labelling in (M) and (N) is displayed in false yellow colour. Higher magnification images of the lobes in (O) and (P) are displayed in a single optical plane, with the red fluorescence and bright-field channels being merged. Scale bars in the whole-grain sections = 500 µm; scale bars in the higher magnification lobes = 100 µm, except (O, P) = 50 µm. Abbreviations: Pb, protein body; Al, aleurone. Starch is indicated by the arrows in (P).

Specific probes for the ω-5 and ω-2 gene sequences were designed to determine their spatial expression patterns at 17 DPA (during the main grain-filling period) by *in situ* hybridization. The results showed that they had similar expression patterns (Fig. 5E–L). Stronger expression was observed in the starchy endosperm at N350 than at N100, with the spatial patterns of expression differing between the two treatments. At N100, expression was observed across the whole starchy endosperm but was greater in the central part than in the peripheral sub-aleurone region. In contrast, much greater expression was observed in the sub-aleurone region compared with the central starchy endosperm at N350. No signal was detected with the sense probe in the starchy endosperm (Supplementary Data Fig. S2A).

### Localization of ω-5 and ω-2 gliadins by immunofluorescence microscopy

Staining of tissue sections at 27 DPA for total proteins with Naphthol Blue Black showed differences between the N100 and N350 treatments in the size and distribution of protein bodies in the starchy endosperm (Fig. 5A–D). In particular, the protein bodies were larger in size and greater in number at N350 and were also concentrated in the outer layers of the starchy endosperm, whereas they were smaller and more evenly distributed across the whole starchy endosperm at N100.

Immunolabelling with the ω-5 monoclonal antibody confirmed that the ω-5 gliadin was mainly located in the outer layers of endosperm, with the outer layers of the lobes being enriched in larger protein bodies and the inner parts in small protein bodies. In contrast, only weak signals were observed in the dorsal region, with the protein bodies showing only light labelling (Fig. 5M–P). Labelling of protein bodies was also more intense in the sample grown at N350 (Fig. 5N, P), in both the lobe and dorsal areas, with the protein bodies also being larger and more numerous (as revealed by staining for total protein). The ω-2 antibody gave high background binding when used for immunolabelling, but the patterns (not shown) were consistent with those revealed using the ω-5 antibody.

## DISCUSSION

The precise numbers of ω-gliadin proteins and genes in wheat have not been determined, with the cloning of ω-gliadin genes and determination of complete sequences still presenting technical challenges. Sabelli and Shewry (1991) used Southern blotting to suggest that bread wheat contained about 15–18 ω-gliadin genes, but did not determine whether these were all expressed or relate them to the proteins separated by electrophoresis (Anderson *et al.*, 2009). A number of authors have identified individual ω-gliadins using N-terminal sequencing and either electrophoresis or reverse-phase HPLC (Kasarda *et al.*, 1983; Masci *et al.*, 1993, 1999; Dupont *et al.*, 2000), with the numbers reported being consistent with the studies reported here. Dupont *et al.* (2011) also identified seven ω-gladins in the bread wheat cultivar Butte 86 by 2-D gel electrophoresis and tandem mass spectrometry, but the proteins were not clearly separated in the first (SDS–PAGE) dimension and it is not known whether they were monomeric or polymeric. We have used a combination of approaches (SDS–PAGE of monomeric and polymeric fractions, western blotting and N-terminal sequencing) to identify and characterize all of the ω-gliadins resolved by SDS–PAGE for six UK wheat cultivars. Five ω-gliadin bands were identified in ‘Hereward’, ‘Istabraq’ and ‘Malacca’, including three ω-5 gliadins (ω-5a, ω-5b and ω-5c) and two bands comprising mixtures of ω-1 and ω-2 gliadins. Only three ω-gliadin bands were identified in ‘Marksman’, ‘Cordiale’ and ‘Xi19’, one ω-5 gliadin and two comprising mixtures of ω-1 and ω-2 gliadins. It can therefore be concluded that these two groups of cultivars contain a minimum of seven and five ω-gliadin proteins, respectively. A polymeric form of ω-5 gliadin (ω-5b) was present in ‘Hereward’, ‘Istabraq’ and ‘Malacca’, and a polymeric form of ω-2 gliadin (ω-2b) was present in all six cultivars. This shows that polymeric forms of ω-gliadins are widely present in modern cultivars of bread wheat, and suggest that further studies of their role in glutenin polymer structure and dough processing properties would be justified.

An increase in the proportion of ω-gliadins with nitrogen fertilization has been reported previously (Moss *et al.*, 1981; Wieser and Seilmeier, 1998; Godfrey *et al.*, 2010; Altenbach *et al.*, 2011) and may result from an imbalance in S availability in relation to N (Moss *et al.*, 1981, 1983). The increased proportion of ω-5 gliadins with nitrogen application reported here may similarly relate to the fact that these proteins have higher contents of nitrogen than the ω-1/2 gliadins (resulting from the presence of 50 % compared with 40 % glutamine, which contains two atoms of nitrogen) (Shewry *et al.*, 2009*a*). The role of ω-gliadins in grain processing is still unclear, with the incorporation of purified proteins into flour reported to result in negative (Uthayakumaran *et al.*, 2001; Fido *et al.*, 1997) or positive (Khatar *et al.*, 2002*a*, *b*) effects on bread making quality. However, these reports studied only monomeric fractions. The large increase in the polymeric ω-5b gliadin may lead to an increase in total glutenin polymers, but this is unlikely to lead to increased quality as the polymeric ω-gliadins generally have single cysteine residues available for the formation of interchain disulfide bonds and hence may act as chain terminators reducing polymer size (Gianibelli *et al.*, 2002). The ω-5 gliadins are also the major components responsible for triggering the most widespread form of food allergy to wheat grain (WDEIA) (Morita *et al.*, 2003; Matsuo *et al.*, 2005).

The increased accumulation of ω-5 and ω-2 gliadins with higher availability of nitrogen was accompanied by increases in related transcripts, determined using real-time RT–PCR and *in situ* RNA hybridization during grain filling (17 DPA). However, that latter approach showed effects of nitrogen on expression patterns within the grain, with ω-gliadin genes being more highly expressed in the central endosperm than in the sub-aleurone cells in the low nitrogen (N100) grain, but expressed more highly in the sub-aleurone cells (especially in the lobes) in the high nitrogen (N350) grain. This difference in distribution was also seen by immunolabelling of grain sections at the end of grain filling (27 DPA). The concentration of proteins, and particularly gliadins, in the sub-aleurone cells of mature grain is well established, based on grain fractionation and microscopy studies (Bradbury *et al.*, 1956; Normand *et al.*, 1965; Kent, 1966; Kent and Evers, 1969; Tosi *et al.*, 2009, 2011; He *et al.*, 2013), but an effect of nitrogen on this distribution has not been reported previously.

The mechanism of regulation of ω-gliadin gene expression has not been studied, but detailed studies of genes encoding C hordein (the homologue of ω-gliadin present in barley) showed that responsiveness to nitrogen (in the form of amino acids) was regulated by a GCN-like motif located 5' upstream of the gene promoter (Müller and Knudsen, 1993). The increased accumulation of ω-gliadins (and other gluten proteins) in the sub-aleurone cells of the starchy endosperm may therefore be due to increased gene expression resulting from transport of surplus nitrogen (amino acids) into these cells under conditions of excess nitrogen supply.

To conclude, we have shown that nitrogen nutrition affects the composition and spatial location of ω-gliadins in the starchy endosperm of wheat grain. These findings suggest that the amounts and compositions of ω-gliadins in grain and food can be manipulated by breeding, agronomy or processing to optimize the functional properties and reduce exposure to allergenic components.

## SUPPLEMENTARY DATA

Supplementary data are available online at www.aob.oxfordjournals.org and consist of the following. Table S1: primers for real-time PCR and RNA probe synthesis. Figure S1: responses to nitrogen application and N-terminal sequences of ω-gliadins in ‘Hereward’ and ‘Cordiale’. Figure S2: negative controls for *in situ* hybridization using the ω-2 gliadin sense probe and the secondary antibody only for immunofluorescence analysis of ω-5 gliadin.

Supplementary Data
