# Supplementary material for: Effects of nitrogen nutrition on the synthesis and deposition of the ω-gliadins of wheat
Source: Ann Bot. 2013 Dec 15;113(4):607–15. doi: 10.1093/aob/mct291 (PMC3936585; doi:10.1093/aob/mct291)
Supplement: Supplementary Data [file supp_113_4_607__index.html]

Effects of nitrogen nutrition on the synthesis and deposition of the ω-gliadins of wheat — Supplementary Data 

# Effects of nitrogen nutrition on the synthesis and deposition of the ω-gliadins of wheat

## Supplementary Data

Supplementary Data

**Files in this Data Supplement:**

- Supplementary Data - Pdf file
