# Supplementary material for: Effects of nitrogen nutrition on the synthesis and deposition of the ω-gliadins of wheat
Source: Ann Bot. 2013 Dec 15;113(4):607–15. doi: 10.1093/aob/mct291 (PMC3936585; doi:10.1093/aob/mct291)

SUPPLEMENTARY DATA

Table S1. Primers for real-time PCR and RNA probe synthesis (underlined sequences for T7 RNA polymerase site).

Gene		Primer sequence	PCR product size	
ω-2	Forward	TGGCCAATGAAATGAACACC	120bp	Real-time PCR
	Reverse	GGTCGGGGTTACACATTATGG		
	Forward	GAATTGTAATACGACTCACTATAGGGCCATATGGGAGTAGTCTTAC	180bp	Sense probe
	Reverse	GAACTTATTTGTCTTTTATTCCCATGTTTGG		
	Forward	CCATATGGGAGTAGTCTTAC	180bp	Antisense probe
	Reverse	GAATTGTAATACGACTCACTATAGGGGAACTTATTTGTCTTTTATTCCCATGTTTGG		
ω-5	Forward	CAAGAACCTTCCCCATACCA	117bp	Real-time PCR
	Reverse	CAACGATGATTCACCCGTCT		
	Forward	GAATTGTAATACGACTCACTATAGGGGGCAAGAACCTTCCCCATACCA	217bp	Sense probe
	Reverse	GAACTTATTTGTCTTTTATTCCCATGTTTGG		
	Forward	GGCAAGAACCTTCCCCATACCA	217bp	Antisense probe
	Reverse	GAATTGTAATACGACTCACTATAGGGGAACTTATTTGTCTTTTATTCCCATGTTTGG		

Fig. S1. Response of ω-gliadins to nitrogen application and N-terminal sequencing in (A) ‘Hereward’ (reported by Shewry *et al.*, 2009*b*) and (B) ‘Cordiale’. Fractions from grain grown at N100, N200, and N350 kg N ha^{-1} are shown in lanes 1, 2 and 3, respectively.

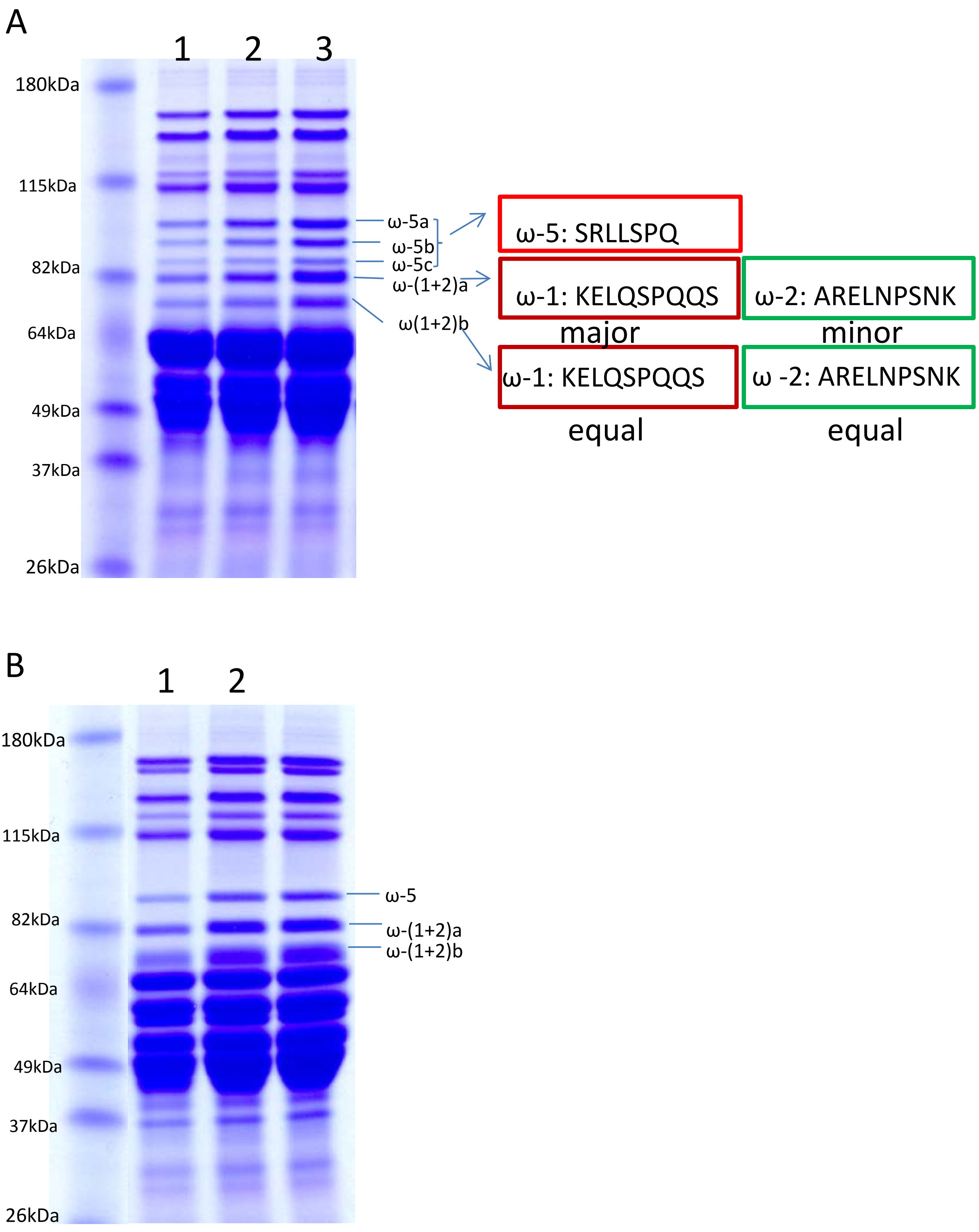

Fig. S2. Negative controls for *in situ* hybridisation using (A) the ω-2 gliadin sense probe and (B) the secondary antibody only for immunofluorescence analysis of ω-5 gliadin. Scale bars: 500 µm.

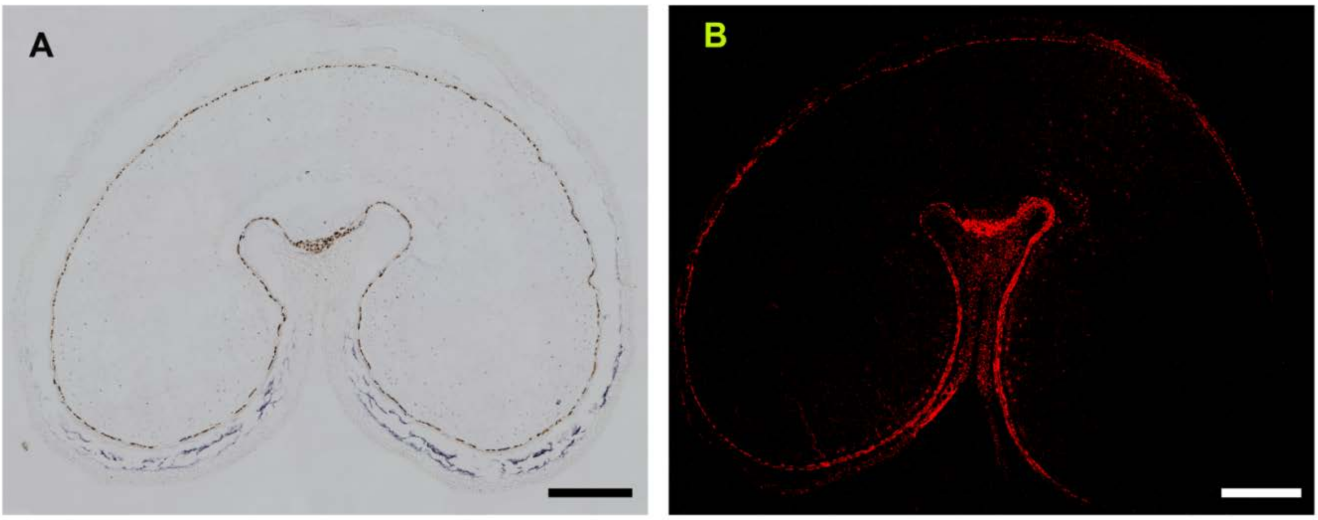

Supplement: Supplementary Data [file supp_mct291_mct291supp.pdf]
